# Statin Drugs Plus Th1 Cytokines Potentiate Apoptosis and Ras Delocalization in Human Breast Cancer Lines and Combine with Dendritic Cell-Based Immunotherapy to Suppress Tumor Growth in a Mouse Model of HER-2^pos^ Disease

**DOI:** 10.3390/vaccines8010072

**Published:** 2020-02-06

**Authors:** Crystal M. Oechsle, Loral E. Showalter, Colleen M. Novak, Brain J. Czerniecki, Gary K. Koski

**Affiliations:** 1 Department of Biological Sciences, School of Biomedical Sciences, Kent State University, Kent, OH 44242, USA; coechsl@bgsu.edu (C.M.O.); cnovak13@kent.edu (C.M.N.); 2 Ohio Attorney General’s Center for the Future of Forensic Science, Department of Biological Sciences, Bowling Green State University, Bowling Green, OH 43403, USA; 3 Department of Biological Sciences, Kent State University, Kent, OH 44242, USA; lshowal1@kent.edu; 4 Department of Breast Oncology, Moffitt Cancer Center, Tampa, FL 33612, USA; Brian.Czerniecki@moffitt.org

**Keywords:** statin, breast cancer, cytokine, apoptosis, Ras, vaccine

## Abstract

A dendritic cell-based, Type 1 Helper T cell (Th1)-polarizing anti-Human Epidermal Growth Factor Receptor-2 (HER-2) vaccine supplied in the neoadjuvant setting eliminates disease in up to 30% of recipients with HER-2-positive (HER-2^pos^) ductal carcinoma in situ (DCIS). We hypothesized that drugs with low toxicity profiles that target signaling pathways critical for oncogenesis may work in conjunction with vaccine-induced immune effector mechanisms to improve efficacy while minimizing side effects. In this study, a panel of four phenotypically diverse human breast cancer lines were exposed in vitro to the combination of Th1 cytokines Interferon-gamma (IFN-γ) and Tumor Necrosis Factor-alpha (TNF-α) and lipophilic statins. This combination was shown to potentiate multiple markers of apoptotic cell death. The combination of statin drugs and Th1 cytokines minimized membrane K-Ras localization while maximizing levels in the cytoplasm, suggesting a possible means by which cytokines and statin drugs might cooperate to maximize cell death. A combined therapy was also tested in vivo through an orthotopic murine model using the neu-transgenic TUBO mammary carcinoma line. We showed that the combination of HER-2 peptide-pulsed dendritic cell (DC)-based immunotherapy and simvastatin, but not single agents, significantly suppressed tumor growth. Consistent with a Th1 cytokine-dependent mechanism, parenterally administered recombinant IFN-γ could substitute for DC-based immunotherapy, likewise inhibiting tumor growth when combined with simvastatin. These studies show that statin drugs can amplify a DC-induced effector mechanism to improve anti-tumor activity.

## 1. Introduction

The Human Epidermal Growth Factor Receptor-2 (HER-2) oncodriver is associated with a poorer prognosis for breast cancer [1,2]. However, the use of HER-2 peptide-loaded IL-12-secreting dendritic cells (DC) for neoadjuvant vaccination against early breast cancer (DCIS; ductal carcinoma in situ) has led to complete pathological responses (pCR; i.e., no remaining disease) in up to 30% of immunized subjects [3,4,5,6]. The vaccine elicited strong Type 1 helper T cell (Th1) immunity, with high levels of CD4 T cells often observed infiltrating areas of disease post-vaccination, and strongly suppressed HER-2 expression in many patients with residual disease [3,4]. Predominance of peritumoral CD4 T cells suggested Th1 cytokines may be responsible for some of the vaccine effect. Indeed, combinations of IFN-γ and TNF-α typically induced high levels of senescence and apoptosis in multiple HER-2-positive (HER-2^pos^) breast and other cancer lines in vitro, with concomitant suppression of HER-family expression, thereby mimicking in vivo observations following DC vaccination [7,8].

In previous vaccine trials, we observed that the majority of pCRs had estrogen receptor (ER)-negative tumors, while ER-positive patients only seldom (5%) displayed pCR [4,5]. This was a concern because nearly 50% of HER-2^pos^ breast cancers also overexpress hormone receptors [9], potentially limiting vaccine utility. We and others propose that combinatorial therapies will be a highly fruitful avenue for improving outcomes for breast cancer [10,11]. Such a strategy, combining anti-estrogen drugs in vitro with Th1 cytokines, greatly enhanced killing of ER-positive breast cancer lines, an approach which was subsequently validated in a clinical trial where anti-estrogen therapy plus vaccination boosted pCR rates of ER-positive subjects from 5% to 30% [6]. By continuing this successful strategy, we should be able to rapidly screen additional candidate drugs in vitro for cooperative effects with Th1 cytokines, and then swiftly translate these findings into pilot clinical studies to determine whether these additions lead to improvements in vaccine efficacy.

Statin drugs are used to treat hypercholesterolemia and taken daily by millions of people with few adverse side effects [12]. In vitro and in vivo evidence suggests that statins may also possess unanticipated anti-cancer properties [13,14,15]. Statins may exert pleiotropic anti-tumor effects by inhibiting the cholesterol biosynthetic (mevalonate) pathway, responsible for both cholesterol production and the generation of lipids that facilitate association of certain molecules to the cell membrane [13,16]. For example, Ras oncogene products rely on the mevalonate pathway for hydrophobic prenyl groups enabling its association with the cytoplasmic face of the plasma membrane, without which Ras cannot signal [17,18]. Simvastatin and fluvastatin are potent lipophilic statin drugs that induce apoptosis in human breast cancer cell lines [19,20,21]. Targeting additional intracellular signaling pathways like Ras, through the use of lipophilic statins, could theoretically enhance tumor cell death in combination with therapeutic vaccination in people. Here, we report that Th1 cytokines work in conjunction with statin drugs to potentiate tumor cell death via apoptosis with the unexpected effect of localizing Ras away from the membrane and into the cytoplasm. Furthermore, statin drugs combined with a DC-based immunotherapy retards tumor progression in a murine model of HER-2^pos^ breast cancer. These findings suggest that statin drugs can act as a kind of “biochemical adjuvant” to enhance the efficacy of anti-cancer immunotherapies that work in part through Th1 immunity, or the effectors thereof. 

## 2. Materials and Methods

### 2.1. Activation of Simvastatin

Activation of simvastatin, which opens the lactone ring, was performed as previously described by others [22]. Briefly, 8 mg of simvastatin was dissolved in 0.2 mL of 100% ethanol (Spectrum, New Brunswick, NJ, USA) and then 0.3 mL of 0.1 M NaOH (Sigma-Aldrich, St. Louis, MO, USA) was added. The solution was heated at 50 °C for 2 h then neutralized with HCl (Sigma-Aldrich, St. Louis, MO, USA) to pH 7.2. The resulting solution was brought to 1 mL with distilled water, and aliquots were stored at −80 °C until use.

### 2.2. Cell Culture

Human breast cancer cell lines SK-BR-3 (RRID: CVCL_0033) and MDA-MB-468 (RRID: CVCL_0419) were obtained from American Type Culture Collection (Rockwell, MA, USA). The HCC1419 (RRID: CVCL_1251) cell line was a gift from Dr. Brian Czerniecki (H. Lee Moffitt Cancer Center), and the MDA-MB-231 (RRID: CVCL_0062) cell line was a gift from Dr. Gail Fraizer (Kent State University). Cell line identity was confirmed by Short Tandem Repeat (STR) profiling. All cell lines were cultured and maintained as described previously [23].

### 2.3. Alamar Blue Assay

Cells were seeded in 96-well culture plates (8 × 10^3^ cells/50 µL per well) and incubated overnight. The next day, cells were treated with Th1 cytokines (ranging from 10 to 20 ng/mL each recombinant human IFN-γ and TNF-α) (Peprotech, Rocky Hill, NJ, USA), or left untreated (controls). Additional treated and untreated groups received statin drugs at concentrations ranging from 0 to 100 µM (Selleckchem, Houston, TX, USA). Cells were incubated for 72 h post-treatment, then treated with Alamar Blue dye and assayed as previously described [23]. 

### 2.4. Trypan Blue Assay

Cells were harvested 72 h post-treatment with Th1 cytokines and statin drugs, stained with Trypan Blue dye, and assessed as described previously [23]. 

### 2.5. Mitochondrial Membrane Potential Assessment (TMRE)

At 24, 48, and 72 h post-treatment with Th1 cytokines and statin drugs, cells were stained with tetramethylrhodamine ethyl ester (TMRE), harvested, and assessed as described previously [23].

### 2.6. Fluorescein Isothiocyanate (FITC)-Annexin V and Propidium Iodide (PI) Staining

Approximately 48 h post-treatment, cells were harvested and resuspended in 50 µL of 1× Binding Buffer (Invitrogen by Thermo Fisher Scientific, Eugene, OR, USA), and 3 µL of FITC-Annexin V (Invitrogen) was added per sample. Samples were incubated for an additional 15 min. Immediately prior to flow cytometry analysis, 0.5 µL of 0.1 mg/mL PI (Invitrogen) was added to each sample. For each sample, 2500 intact/single-cell events were analyzed on the Amnis Flowsight using the 488 nm excitation laser.

### 2.7. CellEvent™ Caspase Activation Assay

Approximately 48 h post-treatment, 1 µL of Caspase 3/7 Green Detection Reagent (Invitrogen) per 1 mL of media was added to each sample, samples were incubated for an additional 30 min. Cells were then harvested and resuspended in 1X Phosphate-Buffered Saline (PBS) (Corning Cellgro, Manassas, VA, USA). For each sample, 5000 intact/single-cell events were analyzed on the Amnis Flowsight using the 488 nm excitation laser, and the shift in fluorescence of the gated single-cell events was used to assess cells with active caspases-3/7. 

### 2.8. Western Blotting

Membrane fractionation was conducted using the Mem-PER™ Plus Membrane Protein Extraction Kit (Cat # 89842, Pierce Biotechnology, Thermo Scientific, Rockford, IL, USA) following the manufacturer’s recommendations. Both the kit buffers (Permeabilization and Solubilization) were supplemented with protease inhibitor cocktail (Pierce) and PhosStop phosphatase inhibitor cocktail (Roche, Mannheim, Germany). Whole-cell lysates were prepared by extracting proteins with RIPA lysis buffer (150 mM sodium chloride, 1.0% Triton X-100, 0.5% sodium deoxycholate, 0.1% sodium dodecyl sulfate, and 50 mM Tris pH 8.0) containing protease inhibitor cocktail and PhosStop phosphatase inhibitor cocktail. Total protein (50 µg) was separated by SDS-polyacrylamide gel electrophoresis and western blotting/signal detection performed as described previously [23]. Primary antibodies included K-Ras (Sigma-Aldrich, St. Louis, MO, USA, #WH0003845M1), β-actin (Millipore, #MAB1501), MAP-ERK ½ (Santa Cruz Biotechnology, Santa Cruz, CA, USA, #sc-514302), phospho MAP-ERK ½ (Cell Signaling, Danvers, MA, USA, #5726S), total AKT (Cell Signaling, Danvers, MA, USA, #9272S), and GAPDH (Cell Signaling, Danvers, MA, USA, #5174S). Secondary antibodies included HRP-conjugated mouse-IgGκ (Santa Cruz, CA, USA, #sc-516102) and mouse anti-rabbit IgG-HRP (Santa Cruz, CA, USA, #sc-2357). 

### 2.9. T-Lymphocyte Functional Studies

For allogeneic Mixed Lymphocyte Reactions (MLRs), human peripheral blood mononuclear cells were obtained from healthy volunteers via leukapheresis after provision of informed written consent, and in accordance with the principals of the Declaration of Helsinki and National Institutes of Health (NIH) guidelines for human subjects, through protocols approved by the Institutional Review Boards of the Cleveland Clinic (08–957) and Kent State University (18–421), and collected cells were separated via countercurrent centrifugal elutriation into monocyte-rich and lymphocyte-rich fractions [24]. Co-cultures of allogeneic activated dendritic cells and lymphocytes derived from elutriated cells were set up as described previously [23] in the presence or absence of 1–10 µM simvastatin, and IFN-γ assessed in co-culture supernatants 72 h later as described previously [23]. For EliSpot assays, human Peripheral Blood Mononuclear Cells (PBMCs) were purchased from CTL Corporation (Shaker Heights, OH, USA) and stimulated in vitro with viral peptide ‘plus’ peptide pool (10 µL/well; CTL corp) or Tetanus Toxoid (2 µg/mL; Sigma) in the presence or absence of 1–10 µM simvastatin in 96-well IFN-γ EliSpot assay plates (CTL corp). After 24 h, incubation plates were developed as per manufacturer’s protocol and analyzed as described previously [23]. 

### 2.10. Generation of Mouse Dendritic Cells

Immature DCs were obtained from the bone marrow of female Balb/c mice as previously described [25]. Briefly, bone marrow was harvested from Balb/c mouse femur and tibia, cultured for six days in RPMI-1640 media (BioWhittaker, Walkersville, MD, USA) supplemented with 10% v/v fetal bovine serum (FBS) (Atlanta Biologicals, Flowery Branch, GA, USA), 100 units/mL of potassium penicillin and 100 µg/mL of streptomycin sulfate (BioWhittaker), 2 mM L-glutamine (BioWhittaker), 1 mM sodium pyruvate (BioWhittaker), 1% non-essential amino acids (BioWhittaker), 30 ng/mL human Flt-3L and 25 ng/mL murine IL-6 (both from Peprotech, Rocky Hill, NJ, USA). On day six, cells were harvested, washed twice in PBS, resuspended in culture media supplemented with 50 ng/mL murine GM-CSF and 10 ng/mL IL-4 (both from Peprotech), and incubated overnight. The next day, the cells were harvested then frozen in FBS (2 × 10^7^ cells) containing 10% DMSO (Sigma-Aldrich, St. Louis, MO, USA). On the day of vaccination, cells were thawed, washed once with PBS to remove DMSO, and then resuspend in culture media containing only 1% FBS and supplemented with murine GM-CSF and IL-4. After 1 h of incubation, the cells were exposed to murine HER-2 peptides (two class II peptides and one class I peptide), incubated for 2 additional hours, activated with 20 ng/mL LPS and 10 ng/mL CpG (both from Invivogen, San Diego, CA, USA), incubated for 2 more hours, harvested, washed three times in PBS, then resuspended in PBS at a concentration of 1 × 10^7^ cells/mL for vaccination.

### 2.11. Therapy Model

#### 2.11.1. Mice

Female Balb/c mice, 10–12 weeks of age, were purchased from Charles River Laboratories (Wilmington, MA, USA). They were maintained in a specific pathogen-free environment in the animal facility at Kent State University in accordance with the NIH guidelines. Experiments were approved by the Institutional Animal Care and Use Committee of Kent State University (protocols 451GK17-15 and 481 GK 19-05).

#### 2.11.2. Tumor Model

The mouse mammary tumor cell line, TUBO (RRID: CVCL_2A33), was a gift from Dr. Wei Zen Wei of Wayne State University, being originally isolated from a spontaneously arising mammary tumor in a rat HER-2 transgenic mouse [26]. TUBO cells were cultured in RPMI-1640 media supplemented with 10% v/v fetal calf serum (FCS; Atlanta Biologicals, Flowery Branch, GA, USA), 100 units/mL of potassium penicillin and 100 µg/mL of streptomycin sulfate (BioWhittaker), 2 mM L-glutamine (BioWhittaker), 1 mM sodium pyruvate (BioWhittaker), and 1% non-essential amino acids (BioWhittaker). Cells were harvested with Cell Dissociation Buffer (Gibco, Grand Island, NY, USA), washed twice with PBS, and then resuspended in PBS for inoculation.

Thirty Balb/c mice were divided into six groups containing five mice per group. On day zero, all mice were subcutaneously inoculated with 2.5 × 10^5^ TUBO cells in 100 µL of PBS. At Day 7 post-implantation, when tumors became palpable, mice were either left untreated (control) or treated with simvastatin (20 mg/kg i.p.); peptide-pulsed DC (1 million DC/treatment/mouse s.q. flanks), IFN-γ (10 µg/mouse i.p.); DCs plus simvastatin, or IFN-γ plus simvastatin. Peptide-pulsed dendritic cells were administered twice weekly over the course of three weeks, for a total of six treatments. Both simvastatin and IFN-γ were given five times per week for three weeks. Calipers were used to measure the total tumor volume (length multiplied by width) every 2–3 days. Treatment was ceased on Day 25, but tumors were monitored until Day 30, when in adherence with our protocol, mice were taken down due to tumor volume.

### 2.12. Statistical Analysis

Experiments were repeated at least three times with consistent results. Values were processed using Microsoft Excel 2016 software and are presented as the mean ± standard error of the mean (SEM). Statistical analyses were performed using one-way ANOVA; *p*-values < 0.05 were considered statistically significant. To identify statistical differences between treatment groups following ANOVA, the Tukey’s Honest Significant Difference (HSD) post-hoc test was used. Statistical analyses were conducted with SigmaPlot v12.0 statistical software (Systat Software Inc., San Jose, CA, USA). 

## 3. Results

### 3.1. Th1 Cytokines Work Cooperatively with Statin Drugs to Suppress Metabolic Activity of Breast Cancer Cells

**Figure 1:**
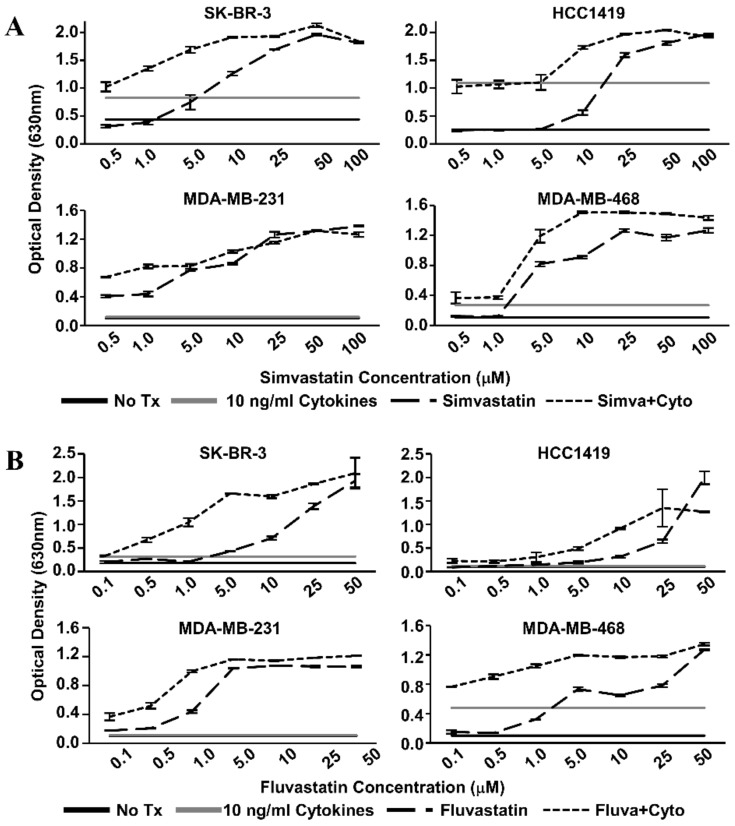
Statin dose–response curves via Alamar Blue dye reduction assay. Human breast cancer cell lines (SK-BR-3, HCC1419, MDA-MB-231, and MDA-MB-468) were treated with increasing concentrations of (**A**) Simvastatin or (**B**) Fluvastatin in the presence (short dash) or absence (long dash) of recombinant Th1 cytokines (Tumor Necrosis Factor-alpha, TNF-α and Interferon-gamma, IFN-γ, 10 ng/mL each) for 72 h. Alamar Blue dye was added and, following color change, the optical density of the dye in the culture supernatants was determined. Optical Density (OD) values of untreated controls (black) and cytokine only treatment (gray) are represented as horizontal lines.

**Figure 2:**
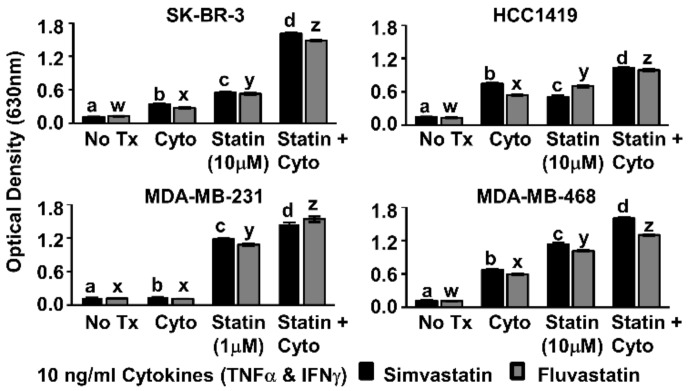
Combination of Th1 cytokines and statin drugs potentiates metabolic suppression in breast cancer lines. SK-BR-3, HCC1419, MDA-MB-231, and MDA-MB-468 human breast cancer cell lines were cultured with no additives (No Tx), treated with recombinant Th1 cytokines (“Cyto” TNF-α and IFN-γ, 10 ng/mL each), statin drugs (Simvastatin or Fluvastatin, 1 µM MDA-MB-231; 10 µM remaining cell lines), or the combination of Th1 cytokines and a statin drug (“Statin + Cyto”). After 72h incubation, Alamar Blue dye was added and, following color change, optical density of culture supernatants was determined. Results displayed are from one representative experiment of at least four trials +/− Standard Error of the Mean (SEM). Letter designations represent Tukey’s Honest Significant Difference (HSD) comparisons: treatments with the same letter designation are not statistically different; when letter designations differ between treatments, the p-value is less than 0.05.

We examined the effects of Th1 cytokines and statin drugs on four human breast cancer cell lines. These included SK-BR-3 and the herceptin-resistant HCC1419 as representative HER-2^pos^ cells, and for comparison, “triple-negative” lines MDA-MB-231 and MDA-MB-468 (Table 1). We restricted our study to ER-negative lines because previous reports identified the ER-positive phenotype as relatively insensitive to statins [27]. Initial dose–response studies (Figure 1) established optimized cytokine/drug concentrations used throughout the study; 10 ng/mL TNF-α, 10 ng/mL IFN-γ, 1 µM simvastatin or fluvastatin (MDA-MB-231 only), and 10 µM simvastatin or fluvastatin (SK-BR-3, HCC1419, and MDA-MB-468). Although each cell line showed variable sensitivity to single agents, all four cell lines showed significantly (*p* < 0.001) less reduction of alamar blue dye (indicating decreased metabolism) when treated with statin drugs and Th1 cytokines simultaneously (Figure 2). This was true for both simvastatin and fluvastatin. Therefore, statin drugs and Th1 cytokines displayed at least additive effects for suppressing cellular metabolism of breast cancer lines.

### 3.2. Combined Treatment Leads to Increased Cell Death

**Figure 3:**
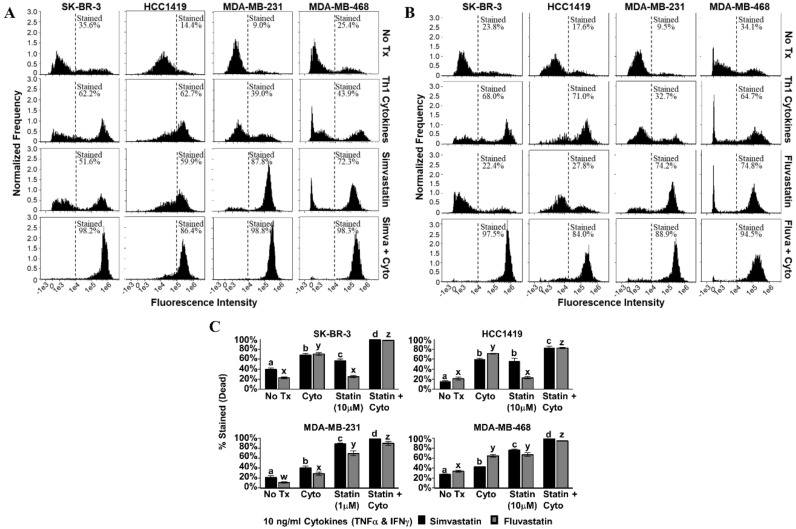
Combination of Th1 cytokines and statin drugs maximize cell death in breast cancer lines. SK-BR-3, HCC1419, MDA-MB-231, and MDA-MB-468 human breast cancer cell lines were cultured with no additives (No Tx), treated with recombinant Th1 cytokines (TNF-α and IFN-γ, 10 ng/mL each), a statin drug (**A**) Simvastatin or (**B**) Fluvastatin (1 µM MDA-MB-231; 10 µM remaining cell lines), or the combination of Th1 cytokines and a statin drug (**A**) “Simva + Cyto” or (**B**) “Fluva + Cyto”. Flow cytometric results displayed in panels A and B are from one representative experiment. (**C**) Graphical interpretation of gated flow cytometric results comparing the percentage of stained events between groups: no additives (No Tx), treated with recombinant Th1 cytokines (TNFα and IFNγ, 10 ng/mL each), a statin drug (Simvastatin or Fluvastatin, 1 µM MDA-MB-231; 10 µM remaining cell lines), or the combination of Th1 cytokines and a statin drug (“Statin + Cyto”). Results displayed are from at least three trials +/− SEM. Letter designations represent Tukey’s HSD comparisons: treatments with the same letter designation are not statistically different; when letter designations differ between treatments, the *p*-value is less than 0.05.

For each cell line tested, combined treatment with Th1 cytokines and simvastatin (Figure 3A) or Th1 cytokines and fluvastatin (Figure 3B) resulted in progressively greater retention of trypan blue dye (rightward shift in histogram) compared to no treatment or treatment with single agents. Statistical analysis of cells (Figure 3C) considered “live” or “dead” by virtue of staining intensity revealed that death induced by combined treatment was significant over single treatments (*p*-values ranged from *p* < 0.001 to *p* = 0.024 depending on cell line and statin combination). The Th1 cytokine–statin combinations in these experiments were highly potent, achieving at least 82% cell death and a maximum of 98%. 

### 3.3. Combined Treatment Results in Higher Frequency of Apoptotic Cell Death

**Figure 4:**
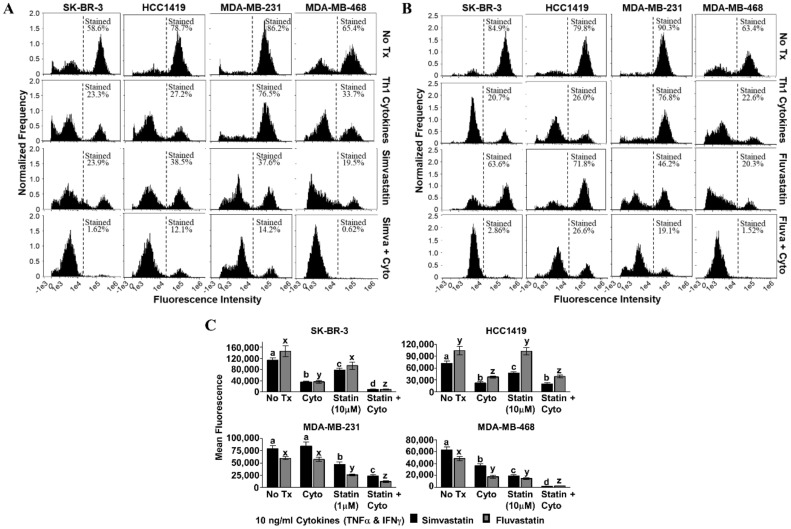
Statin drugs plus Th1 cytokines maximize mitochondrial membrane depolarization as assessed by tetramethylrhodamine ethyl ester (TMRE) staining. SK-BR-3, HCC1419, MDA-MB-231, and MDA-MB-468 human breast cancer cell lines were cultured with no additives (No Tx), treated with recombinant Th1 cytokines (TNF-α and IFN-γ, 10 ng/mL each), a statin drug (**A**) Simvastatin or (**B**) Fluvastatin (1 µM MDA-MB-231; 10 µM remaining cell lines), or the combination of Th1 cytokines and a statin drug (**A**) “Simva + Cyto” or (**B**) “Fluva + Cyto” for approximately 48 h. Flow cytometric results displayed in panels A and B are from one representative experiment. (**C**) Statistical analysis of flow cytometry results comparing the mean channel fluorescent intensity between groups: no additives (No Tx), treated with recombinant Th1 cytokines (TNF-α and IFN-γ, 10 ng/mL each), a statin drug (Simvastatin or Fluvastatin, 1 µM MDA-MB-231; 10 µM remaining cell lines), or the combination of Th1 cytokines and a statin drug (“Statin + Cyto”). Results displayed are from at least five trials +/− SEM. Letter designations represent Tukey’s HSD comparisons: treatments with the same letter designation are not statistically different; when letter designations differ between treatments, the *p*-value is less than 0.05.

We next sought to determine whether there was evidence that cell death proceeded through an apoptotic mechanism. Our criterion contained a set of features associated with apoptosis including mitochondrial depolarization, characteristic changes in cell membranes, and activation of caspase 3/7. In the tetramethylrhodamine ethyl ester (TMRE) assay, dying cells cannot sequester the fluorescent dye in their mitochondria, and so histograms shift left with death (simvastatin, Figure 4A; fluvastatin, Figure 4B). In three of the four cell lines, significantly less fluorescence was observed when statin drugs were combined with Th1 cytokines (*p*-values ranged from *p* < 0.001 to *p* = 0.046, depending on cell line and statin combination) compared with either treatment alone; the exception being HCC1419, in which no significant difference was observed for dual treatment. For both the HCC1419 and SK-BR-3 cell lines (Figure 4C, top panels), it appears that the Th1 cytokines were responsible for the bulk of the mitochondrial transmembrane potential loss. The addition of the statin drug had little to no effect over cytokines alone on the HCC1419 cell line (*p* ≥ 0.948), but the combination did cause a statistically significant, yet modest, decrease in mitochondrial transmembrane potential for the SK-BR-3 cell line (Th1 cytokines with simvastatin, *p* = 0.011; Th1 cytokines with fluvastatin, *p* < 0.001). Treatment with Th1 cytokines had minimal effect on MDA-MB-231 cells (*p* ≥ 0.929) compared with no treatment (Figure 4C, bottom left panel). However, this cell line demonstrated strongly diminished mitochondrial transmembrane potential in response to either statin drug, simvastatin (*p* = 0.005) or fluvastatin (*p* < 0.001). When the statin drugs were combined with Th1 cytokine treatment, there was nonetheless a significantly enhanced effect (*p* < 0.001). Treatment of MDA-MB-468 cells with Th1 cytokines (Figure 4C, bottom right panel) caused a statistically significant, yet modest suppression of mitochondrial transmembrane potential (*p* < 0.001). Statin treatment alone had a similar effect (*p* < 0.001). In contrast, when both treatments were combined, cellular mitochondrial transmembrane potential was suppressed dramatically (*p* < 0.001).

**Figure 5:**
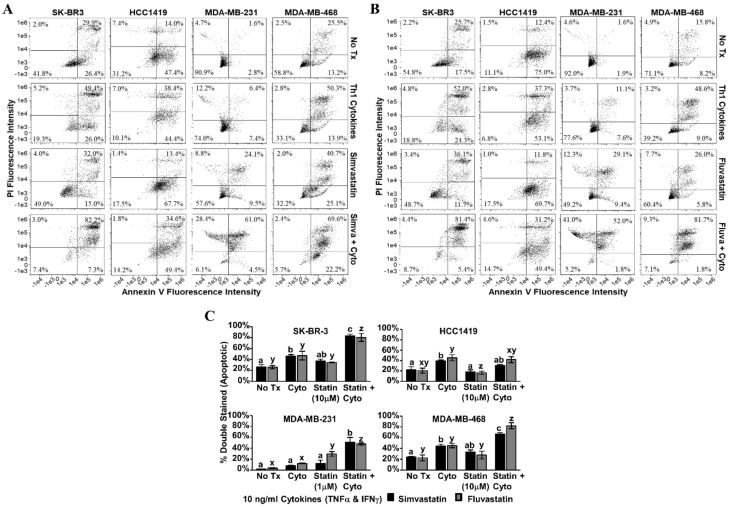
Statin drugs plus Th1 cytokines maximize apoptotic markers in breast cancer lines. SK-BR-3, HCC1419, MDA-MB-231, and MDA-MB-468 human breast cancer cell lines were cultured with no additives (No Tx), treated with recombinant Th1 cytokines (TNF-α and IFN-γ, 10 ng/mL each), a statin drug (**A**) Simvastatin or (**B**) Fluvastatin (1 µM MDA-MB-231; 10 µM remaining cell lines), or the combination of Th1 cytokines and a statin drug (**A**) “Simva + Cyto” or (**B**) “Fluva + Cyto”. Flow cytometric results displayed in panels A and B are of one representative experiment from 3 separate trials with each drug. (**C**) Statistical analysis of composite results for proportions of double-staining cell events between groups: no additives (No Tx), treated with recombinant Th1 cytokines (TNF-α and IFN-γ, 10 ng/mL each), a statin drug (Simvastatin or Fluvastatin, 1 µM MDA-MB-231; 10 µM remaining cell lines), or the combination of Th1 cytokines and a statin drug (“Statin + Cyto”). Results displayed are from three trials +/− SEM. Letter designations represent Tukey’s HSD comparisons: treatments with the same letter designation are not statistically different; when letter designations differ between treatments, the *p*-value is less than 0.05.

Annexin V/PI staining was performed on the second day post-treatment (Figure 5). Dot plot analysis revealed that for SK-BR-3, MDA-MB-231 and MDA-MB-468, treatment with Th1 cytokines plus statin drugs (simvastatin Figure 5A; fluvastatin Figure 5B) resulted in greater numbers of double-stained (i.e., apoptotic) cells compared with single treatments (Figure 5C). For these three lines, these differences were statistically significant (p-values ranging from *p* < 0.001 to *p* = 0.013, depending on line and statin drug). Only HCC1419 cells did not show enhanced apoptosis with dual treatment by this assay. 

**Figure 6:**
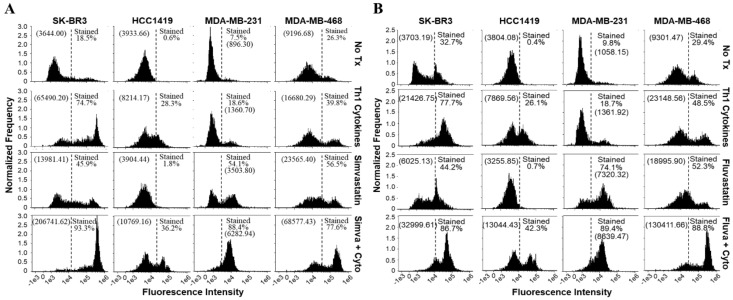
Cells treated with a statin drug and Th1 cytokines demonstrate enhanced activation of caspase 3/7. SK-BR-3, HCC1419, MDA-MB-231, and MDA-MB-468 human breast cancer cell lines were cultured with no additives (No Tx), treated with recombinant Th1 cytokines (TNF-α and IFN-γ, 10 ng/mL each), a statin drug (**A**) Simvastatin or (**B**) Fluvastatin (1 µM MDA-MB-231; 10 µM remaining cell lines), or the combination of Th1 cytokines and a statin drug (**A**) “Simva + Cyto” or (**B**) “Fluva + Cyto” and stained with CellEvent™ fluorescent caspase substrate. The experiment was repeated at least three times; the displayed flow cytometric results are from one representative experiment. The percentage of stained events, as well as the geometric mean channel fluorescence (in parentheses), is annotated on each histogram.

The CellEvent™ assay for detection of activated caspases 3 and 7 was also performed on the second day post-treatment. Here, caspase 3/7 activity in cells undergoing apoptosis alters a cell-permeable substrate which imparts fluorescence to the cells, shifting the histogram curve to the right. Caspase-3/7 activity was clearly enhanced with dual treatment compared with either Th1 cytokines or statins alone (simvastatin Figure 6A; fluvastatin Figure 6B).

### 3.4. Th1 Cytokines and Statin Drugs Affect K-Ras Membrane Localization and Influence Downstream Signaling Targets

**Figure 7:**
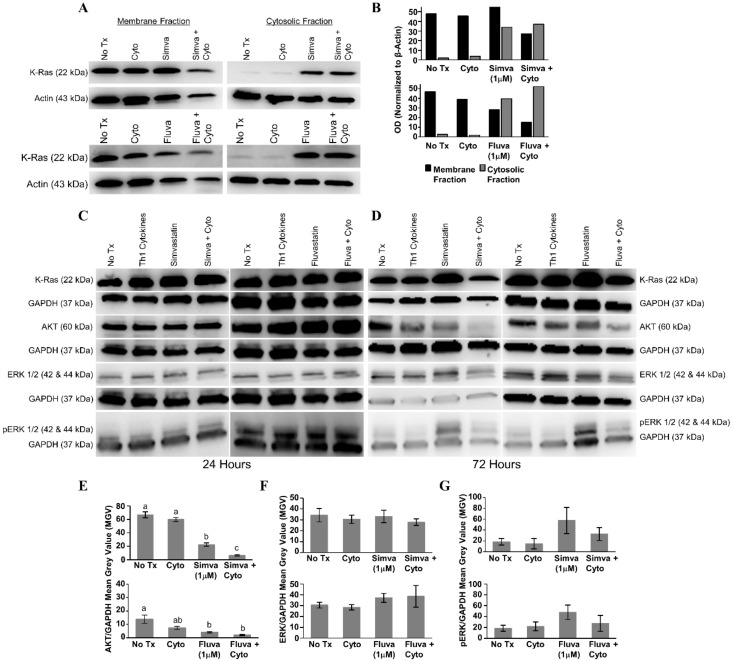
Combined treatment with Th1 cytokines and statin drugs potentiates K-Ras delocalization from membrane. MDA-MB-231 human breast cancer cells were cultured with no additives (No Tx), treated with recombinant Th1 cytokines (TNF-α and IFN-γ, 10 ng/mL each), a statin drug (Simvastatin or Fluvastatin, 1 µM), or the combination of Th1 cytokines and a statin drug (“Simva + Cyto” or “Fluva + Cyto”) for 24 h. Membrane proteins were separated from cytosolic proteins and the distribution of the K-Ras protein was analyzed by Western blot. (**A**) Representative Western blot images and (**B**) corresponding semi-quantitative densitometry analysis of K-Ras normalized to β-actin loading control. Representative Western blot images of whole-cell K-Ras, AKT, ERK ½, and phospho-ERK ½ after (**C**) 24 and (**D**) 72 h of treatment. Semi-quantitative densitometry analysis of the (**E**) GAPDH-normalized total AKT, (**F**) GAPDH-normalized total ERK-1/2, and (**G**) GAPDH-normalized phosphorylated ERK-1/2. All Western blot experiments were repeated at least three times. Displayed graphical results in panels (**E**), (**F**), and (**G**) are from at least three trials +/− SEM. Letter designations in panel (**E**) represent Tukey’s HSD comparisons: treatments with the same letter designation are not statistically different; when letter designations differ between treatments, the *p*-value is less than 0.05.

After 24 h of treatment with statin drugs, Th1 cytokines, or both, the presence of K-Ras in the membrane and cytosolic fractions of MDA-MB-231 cells was assessed (Figure 7A). As illustrated in Figure 7B, densitometry analysis showed that K-Ras level in the membrane fraction (black bars) was the lowest for both simvastatin (upper panel) and fluvastatin (lower panel) when drugs were combined with Th1 cytokines. Conversely, K-Ras levels in the cytoplasm (gray bars) tended to be highest with dual treatment. Yet, we detected no evidence of change in the overall levels of K-Ras from unfractionated cells (Figure 7C,D), suggesting that treatment with cytokines plus drug was causing a redistribution of K-Ras but not its destruction.

When no significant changes in the levels of downstream effectors of Ras were seen 24 h post-treatment (Figure 7C), lysates were examined following 72 h of treatment (Figure 7D). Treatment with Th1 cytokines alone demonstrated a modest decrease in total AKT (simvastatin series, *p* = 0.41; fluvastatin series, *p* = 0.096); there was a significant decrease in total AKT in response to either statin drug (simvastatin, *p* < 0.001; fluvastatin, *p* = 0.015); and when the statin drugs were combined with Th1 cytokines, the decrease in total AKT was further enhanced (Th1 cytokines with simvastatin, *p* < 0.001; Th1 cytokines with fluvastatin, *p* = 0.005) (Figure 7E). No statistically significant change in the amount of total ERK was observed (Figure 7F), yet while also not achieving statistical significance (simvastatin series, *p* = 0.232; fluvastatin series, *p* = 0.311), consistent trends were observed including enhanced phosphorylation with statins alone which were subsequently diminished when combined with Th1 cytokines (Figure 7G).

### 3.5. Simvastatin Does Not Interfere with T Cell Function and Potentiates Anti-Tumor Activity When Combined with DC-Based Immunotherapy in a Mouse Model of HER-2^pos^ Breast Cancer

**Figure 8:**
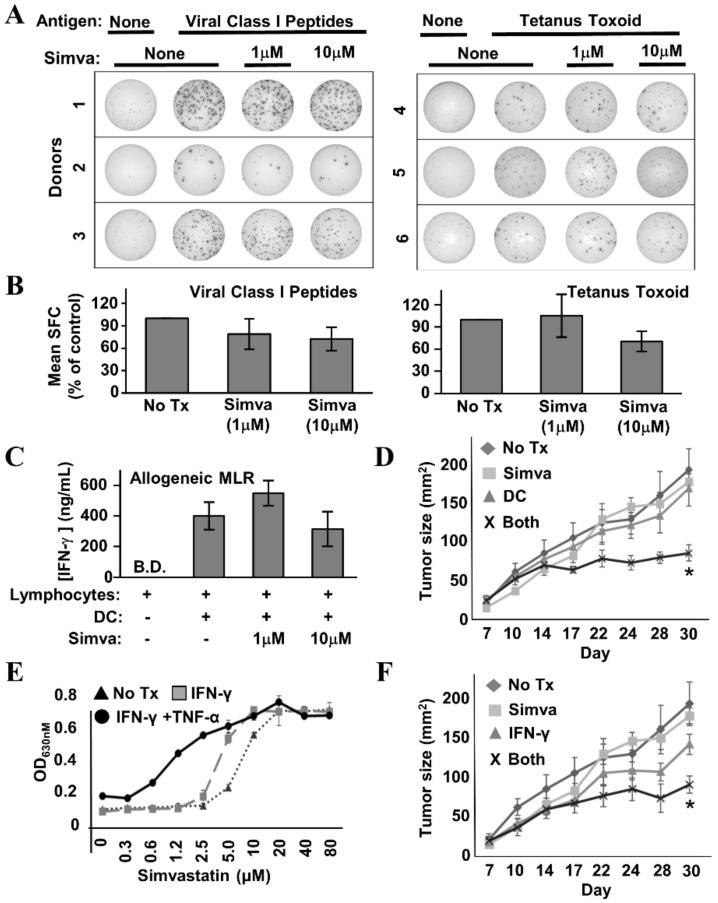
Simvastatin does not interfere with T cell function and enhances effects of immunotherapy in a mouse model of HER-2^pos^ breast cancer. (**A**) Example IFN-γ EliSpot wells from individual healthy donor Peripheral Blood Mononuclear Cells (PBMCs) stimulated with a mixture of common viral peptide recall antigens (left panel) or tetanus toxoid (right panel) in the presence or absence of simvastatin (“Simva”; 1–10 µM). (**B**) Composite analysis of each of the 3 donors for viral recall peptides (left panel) and 3 donors for tetanus toxoid (right panel). Statistical analysis by one-way ANOVA indicated no significant difference between simvastatin-treated and untreated groups. (**C**) IFN-γ ELISA analysis of 72 h culture supernatants from allogeneic Mixed Leukocyte Reactions (MLRs) where activated dendritic cells (DC) and lymphocyte-rich elutriation fractions were co-cultured at 1:40 stimulator:responder ratios in the presence or absence of 1–10 µM simvastatin. Data displayed represents the mean IFN-γ production from seven unique allogeneic DC:lymphocyte pairings. Statistical analysis by one-way ANOVA indicated no significant difference between simvastatin-treated and untreated groups (B.D. = below detection). (**D**) TUBO-bearing Balb/c mice, 7 days after implantation, were either left untreated (“No Rx”) or treated with Simvastatin (“Simva”), peptide-pulsed DC-based therapy (“DC”), or given combined treatment with simvastatin plus peptide-pulsed DCs (“Both”). Tumor size was measured periodically with calipers during treatment. Tumor size denoted as average area +/− SEM. Statistical significance is indicated by the asterisk (*). (**E**) TUBO cells were cultured in vitro with IFN-γ plus TNF-α, IFN-γ only, or no treatment in the presence of increasing concentrations of Simvastatin for 72 h, then assessed for metabolic activity by the Alamar Blue dye assay. (**F**) TUBO-bearing Balb/c mice, 7 days after implantation, were either left untreated (“No Rx”) or treated with Simvastatin (“Simva”), Interferon-γ (“IFN-γ”) or given combined treatment with simvastatin plus IFN-γ (“Both”). Tumor size was measured periodically with calipers during treatment. Tumor size denoted as average area +/− SEM. Statistical significance is indicated by the asterisk (*).

If the intent is to improve immune response rates by using statins in conjunction with Th1-polarizing vaccine therapy, the drugs must not interfere severely with T cell function—particularly their ability to produce effector cytokines. Hence, we examined the capacity of healthy donor lymphocytes to secrete IFN-γ in response to a number of stimuli provided in the presence or absence of simvastatin. IFN-γ EliSpot analysis of healthy donor Peripheral Blood Mononuclear Cells (PBMCs) stimulated with a pool of common viral peptide recall antigens (Figure 8A,B, left panels) or tetanus toxoid (Figure 8A,B, right panels) showed no more than mild simvastatin-induced diminution of spot-forming cells, which did not reach statistical significance. Likewise, in the allogeneic Mixed Leukocyte Reaction (MLR) (Figure 8C), IFN-γ levels were only slightly reduced at the highest concentration of simvastatin (10 µM), and as with the Elispot analysis, the differences were not statistically significant. We therefore conclude that simvastatin does not severely inhibit the capacity of T cells to respond to antigen and secrete IFN-γ. 

To determine whether the in vitro effects of simvastatin and cytokines could be replicated in the setting of active therapeutic vaccination, TUBO murine breast carcinoma cells, which overexpress the rat neu homolog, were implanted into the region of the fat pad of the breast in female Balb/c mice. Seven days later, when tumors were palpable, tumor-bearing mice were divided into four groups. The first received no treatment (control). The second received rat HER-2/neu peptide-pulsed DC immunotherapy twice weekly via subcutaneous flank injection over the course of three weeks. The third group received three 5-day cycles of simvastatin over the same time period as the DC group. The final group received both therapies concurrently. Tumor growth was monitored periodically from the onset of therapy (Figure 8D). It was clear from the growth kinetic curves that single treatments were virtually superimposable on the untreated group. Only the group treated with both DC-based immunotherapy *and* simvastatin showed obvious suppressed growth, and statistical analysis of tumor sizes on the final day of the study showed dual treatment was significant over all other groups (dual treatment versus no treatment, *p* = 0.0007; dual treatment versus simvastatin alone, *p* = 0.02; dual treatment versus dendritic cells alone, *p* = 0.03).

If the DC therapy’s contribution to the anti-tumor activity of dual-treatment therapy occurs substantially through the induction of Th1 soluble factors, then it should be possible substitute vaccination with the in vivo administration of recombinant cytokines, and still see the original therapeutic effect when supplied in combination with simvastatin. To test this hypothesis, we first confirmed that TUBO cells were sensitized to cytokine-mediated cell death by the presence of simvastatin (Figure 8E). We treated cultured TUBO cells with either IFN-γ plus TNF-α, or IFN-γ alone in the presence of increasing concentrations of simvastatin. As concentrations of simvastatin rose, alamar blue analysis showed a strong leftward shift of the dose–response curve when both Th1 cytokines were present, indicating the drug indeed sensitized TUBO cells to Th1 cytokine-mediated cell death. When the only cytokine was IFN-γ, there was likewise a leftward shift, albeit not as pronounced. 

Despite the lower magnitude of anti-tumor activity seen in vitro when IFN-γ was the only Th1 cytokine, for the in vivo study, we chose to omit TNF-α, due to its reputation of unacceptably high toxicity when administered systemically. For this experiment, we chose a similar experimental design as before, but instead of DC-based immunotherapy, mice received 10 μg doses of murine recombinant IFN-γ administered intraperitoneally on the same days they received simvastatin. We found that IFN-γ alone seemed to slightly retard tumor outgrowth (Figure 8F), but this retardation was not significantly different from no treatment. In contrast, IFN-γ plus simvastatin significantly suppressed tumor growth compared with no treatment (*p* = 0.003), and to a degree highly similar to that seen with DC-based immunotherapy plus simvastatin (Figure 8D). This study shows that simvastatin enhances vaccine effects in vivo, and furthermore is consistent with a cytokine-based immune effector mechanism for combined activity. 

## 4. Discussion

The use of statin drugs is associated with increases in apoptosis and lower recurrence rates in breast cancer [14,15,19,34]. In addition to breast cancer, epidemiological studies have also suggested anti-cancer effects of statins in prostate, colorectal, and endometrial cancer. Overall, these observations have prompted suggestions of clinical trials incorporating statin drugs with current treatment regimens [13]. Such actions are particularly timely, since it is being increasingly recognized that the 10–15 year development cycle and billion-plus dollar investment required to bring novel drugs to market is probably unsustainable in the framework of current national and global healthcare [35]. Consequently, the concepts of “drug repurposing” and “drug repositioning” have been advanced [36]. “Repurposing” describes a recommended strategy of identifying anti-cancer activity in existing FDA-approved drugs, not originally developed for treatment of malignancies, to speed deployment and reduce costs of improved therapies. Similarly, “repositioning” refers to identifying a new setting or context that derives improved activity from an existing drug. We not only provide justification for the repurposing and repositioning of simvastatin as an anti-cancer agent, but also suggest this drug as a representative of a new category of compounds that might be called “biochemical adjuvants”. Whereas traditional vaccine adjuvants work by enhancing the magnitude or quality of immune responses during the priming phase, a biochemical adjuvant, as we use this term, does not alter immunity per se, but instead work by sensitizing the target to the immunotherapy induced effectors of immunity.

Because we have developed a DC-based vaccine that induces strong Th1 immunity in humans, we began our studies by examining the contributory effects of Th1 cytokines (the primary effectors of these lymphocytes) and two lipophilic statin drugs on diverse breast cancer lines. We indeed found that statin drugs work with Th1 cytokines to potentiate apoptosis in breast cancer cells. For most tested lines, Th1 cytokines and statin drugs each separately contributed substantial activity. The exception was MDA-MB-231 cells. This line was relatively resistant to effects of Th1 cytokines while showing more sensitivity to statin drugs alone. Nonetheless, combination of Th1 cytokines with either of two structurally distinct lipophilic statin drugs consistently and significantly maximized markers of cell death. Relative statin sensitivity of MDA-MB-231 is probably explained by constitutively activated Ras, and has been reported previously [27]. Consequently, this line is probably more dependent (oncogene addiction) on Ras signaling than the other tested lines. 

There were two unexpected findings. The first was that Th1 cytokines and statin drugs worked cooperatively to delocalize K-Ras from the membrane fraction and into the cytoplasm. The second was the increased phosphorylation of ERK in statin-treated cells. Though constitutively activated in MDA-MB-231 cells, Ras must still localize in the plasma membrane to function [17,18,37]. Membrane association is obtained in two ways: through a lipid adduct (such as farnesylation), synthesis of which is dependent upon activity of the mevalonate pathway, and also through the second signal located in the linker domain of the protein’s C-terminal hypervariable region (in the case of K-Ras, a polybasic domain, which interacts with inner-leaflet phosphatidylserine residues) [38,39]. However, since ERK phosphorylation is associated with K-Ras activity, why does statin treatment increase downstream ERK phosphorylation rather than decrease it, and how can Th1 cytokines contribute to statin-induced Ras displacement?

To explain these observations, it is important to note that the inhibition of the mevalonate pathway by statins inhibits cholesterol synthesis, thus lowering membrane cholesterol content [40]. Cholesterol is critical for maintaining microdomain structures known as caveolae, which sequester membrane lipids, including phosphatidylserine. In fact, phosphatidylserine sequestration by caveolae leaves less than 40% of inner-leaflet phosphatidylserine available for interaction with signaling molecules such as Ras. Loss of membrane cholesterol would disrupt caveolae and allow redistribution of phosphatidylserine in the inner leaflet membrane, promoting K-Ras nanoclustering and the formation of effective signaling complexes leading to downstream phosphorylation of ERK [18,41,42,43]. So, even if statin drugs inhibit localization of Ras to the membrane, any of the molecules already in the membrane could likely signal more efficiently, accounting for the higher levels of ERK phosphorylation seen with statin treatment alone. 

As for the role of Th1 cytokines in enhancing K-Ras delocalization from the membrane fraction, the cytokines IFN-γ and TNF-α have been shown by others to affect lipid metabolism and membrane composition. More specifically, the cytokines have been shown to increase the presence of saturated fatty acyl groups on membrane components, while decreasing unsaturated fatty acyl groups, leading to overall lowered membrane fluidity [44,45]. Significantly, K-Ras has been shown to optimally associate with membranes when the bilayer is more loosely packed (i.e., more unsaturated fatty acyl groups) [46,47]. Consequently, Th1 cytokines would be expected to induce membrane properties less favorable for Ras association. In addition, we show that the Th1 cytokines, as a consequence of apoptosis induction, cause the appearance of phosphatidylserine on the outer leaflet of the plasma membrane (these are presumably flipped from the inner leaflet during apoptosis). Therefore, yet another important anchoring molecule for Ras is diminished in cells treated with both statins and Th1 cytokines. Fully testing this proposed mechanism will require a detailed study of changes in membrane composition and biophysical properties induced by combinations of statin drugs and Th1 cytokines. 

Because the PI3K/PBK/AKT intracellular signaling pathway also lies downstream of Ras, and promotes aspects of cell growth, survival, motility and resistance to apoptosis [48], we initially anticipated seeing attenuated phosphorylation in members of this pathway, owing to presumed losses in upstream Ras activity. Instead, we observed a trend toward loss of total AKT expression evident by 72 h post-treatment (Figure 7D,E). This is perhaps not surprising given that we had previously reported selective loss of HER-family receptors in multiple breast cancer lines undergoing Th1 cytokine-induced apoptosis [8]. It is likely that the expression of multiple components of growth factor signaling pathways are particularly sensitive to negative regulation during apoptosis as a means of limiting conflicting growth/death signals as apoptosis proceeds. 

Perhaps of greatest interest was our demonstration that simvastatin combined with an IL-12-secreting DC-based therapy significantly retarded tumor growth kinetics while single treatments did not. We predicted this would be the case, since both Th1 immunity and statin use has been associated with better outcomes for breast cancer [49,50]. Even a single parenterally supplied Th1 cytokine (IFN-γ) showed substantial increased activity in combination with simvastatin in the mouse model (Figure 8F), consistent with the notion that Th1 cytokines are major effector mechanisms of DC immunotherapy induced responses in this model, and furthermore suggesting a non-cell-based immunotherapy (i.e., a recombinant cytokine) that can be used in combination with simvastatin to achieve an anti-tumor effect. It is tempting to speculate that the subset of breast cancer patients who benefit from statins may be those who already have substantial associated anti-tumor Th1 immunity, insufficient to provide protection on its own, but adequate to suppress cancer recurrence when combined with the drug. It should be noted that the mouse model used in these studies, as a proof-of-principle, involved providing treatment in the setting of a progressively growing tumor, and the immunotherapy (as constituted in the study) in combination with statin drugs, though substantially slowing tumor growth, was not actually curative. This model may, however, supply a basis for optimization that may achieve improvements in therapeutic activity. 

## 5. Conclusions

Th1 cytokines, when combined with lipophilic statin drugs, enhance cell death in cultured HER-2^pos^ and triple-negative breast cancer lines through an apparent apoptotic mechanism. This treatment combination also led to maximized delocalization of the K-Ras oncodriver from the plasma membrane and into the cytoplasm by 24 h exposure, as well as a general loss of AKT expression by 72 h—both consequences consistent with losses in cell viability. HER-2 pulsed dendritic cells, as well as recombinant IFN-γ, when combined with statins, suppressed the outgrowth of tumors in a murine model of HER-2^pos^ breast cancer. These findings provide justification for trials with combinations of immunotherapies promoting strong Th1 immunity, such as a Th1-polarizing DC vaccine, with well-tolerated statin drugs to improve outcomes in breast cancer. Possible test settings include treatment after surgical resection of disease to prevent recurrence, as well as neoadjuvant treatment of high-risk HER-2^pos^ DCIS patients to minimize chances of recurrence, invasion, and metastasis. Combining select low-cost, low-risk targeted drugs with immunotherapies to improve efficacy is a promising strategy that probably warrants further investigation. 

## Figures and Tables

**Table 1:** Properties of the human breast cancer cell lines subjected to treatment.

	SK-BR-3	HCC1419	MDA-MB-231	MDA-MB-468
Subtype [28,29]	Luminal Subtype	Luminal Subtype	Basal B Subtype	Basal A Subtype
Source [28]	Pleural Effusion	Primary Tumor	Pleural Effusion	Pleural Effusion
Tumor Type [28]	Adenocarcinoma	Ductal Carcinoma	Metastatic Adenocarcinoma	Metastatic Adenocarcinoma
Estrogen Receptor [28,29,30]	-	-	-	-
Progesterone Receptor [28,29,30]	-	-	-	-
EGFR * [30]	+2	-	+1	+3
HER-2 * [28,29,30]	+3	+1	-	-
HER-3 * [8,29,31]	Moderate	Moderate/High	Low/Absent	Moderate
Ras Mutant [32,33]	Wild Type	Wild Type	mut-K	Wild Type

* EGFR (Epidermal Growth Factor Receptor), HER-2 (Human Epidermal Growth Factor Receptor-2), HER-3 (Human Epidermal Growth Factor Receptor-3).
